# Enhancing prompt perception in dementia: a comparative study of mixed reality cue modalities

**DOI:** 10.3389/fspor.2024.1419263

**Published:** 2024-08-09

**Authors:** Shital Desai, Rupsha Mutsuddi, Arlene J. Astell

**Affiliations:** ^1^ Department of Computational Arts, School of Arts Media Performance and Design, York University, Toronto, ON, Canada; ^2^ Social and Technological Systems (SaTS) Lab, School of Arts Media Performance and Design, York University, Toronto, ON, Canada; ^3^ Department of Design, School of Arts Media Performance and Design, York University, Toronto, ON, Canada; ^4^ Department of Occupational Sciences & Occupational Therapy, University of Toronto, Toronto, ON, Canada; ^5^ Department of Psychiatry, University of Toronto, Toronto, ON, Canada; ^6^ Psychology Department, Northumbria University, Newcastle, United Kingdom; ^7^ KITE Research Institute, University Health Network, Toronto, ON, Canada

**Keywords:** cues, prompts, interaction modality, dementia, active living, perception and action, mixed reality technology, gerontology

## Abstract

**Introduction:**

Dementia impacts millions worldwide and is challenging individuals' ability to engage in daily activities. Active living is crucial in mitigating dementia's neurodegenerative effects, yet people with dementia often struggle to initiate and complete tasks independently. Technologies offer promising solutions to engage people with dementia in activities of active living and improving their quality of life through prompting and cueing. It is anticipated that developments in sensor and wearable technologies will result in mixed reality technology becoming more accessible in everyday homes, making them more deployable. The possibility of mixed reality technologies to be programmed for different applications, and to adapt them to different levels of impairments, behaviours and contexts, will make them more scalable.

**Objective:**

The study aimed to develop a better understanding of modalities of prompts that people with dementia perceive successfully and correctly in mixed reality environments. It investigated interactions of people with dementia with different types of visual (graphics, animation, etc.) and sound (human voice, tones, etc.) prompts in mixed reality technologies.

**Methods:**

We used the Research through Design (RtD) method in this study. This paper describes the findings from the user research carried out in the study. We conducted observation studies with twenty-two people with dementia playing games on off-the-shelf mixed reality technologies, including both Augmented Reality (HoloLens, ArKit on iPhone) and Augmented Virtuality (Xbox Kinect and Osmo) technologies. The interactions with the technologies during the gameplay were video recorded for thematic analysis in Noldus Observer XT (version 16.0) for successful and correct perception of prompts.

**Results:**

A comparison of the probability estimates of correct perception of the prompts by people with dementia suggests that human voice, graphic symbols and text are the most prominently perceived modalities of prompts. Feedback prompts for every action performed by people with dementia on the technology are critical for successful perception and should always be provided in the design.

**Conclusion:**

The study has resulted in recommendations and guidelines for designers to design prompts for people with dementia in mixed-reality environments. The work lays the foundation for considering mixed reality technologies as assistive tools for people with dementia, fostering discussions on their accessibility and inclusive design in technology development.

## 1 Introduction

According to Alzheimer's Diseases International, there are over 55 million people worldwide living with dementia, and the projected number of people with dementia is expected to rise to 139 million by 2050. Dementia affects one in 20 people over the age of 65 and one in 5 over the age of 80 (1). The progression of dementia can severely compromise people with Dementia's ability to participate in daily activities such as reading and cooking and engaging in forms of physical activity such as exercise and sports. People with dementia find it difficult to sequence tasks in an activity, making it difficult for them to engage in day to day life (2). Research has shown that active living—including physical, social, mental, emotional and spiritual activities can slow down the neurodegenerative effects of dementia and have significant long-term benefits on cognition and dementia progression (3, 4). However, people with dementia are less engaged in activities of active living than cognitively healthy people due to social, psychological, and physiological reasons (5, 6). One of the primary reasons for the lack of participation in these activities is that people with dementia have trouble initiating the steps and micro steps in an activity and stringing them together to complete the activity (7–10). People with dementia can easily lose track of where they are within an activity, forget which step they are on and fail to get to the last step of the activity successfully. However, people with dementia can complete each of the steps and micro steps in the activity independently, separate from the activity (11).

Technologies can provide the required prompts to people with dementia to engage and participate in active living such as cooking, grocery shopping, laundry, exercise and sports without losing track, resulting in a good Quality of Life (QoL) (9, 12–15). However, these technologies should ensure independence and autonomy of people with dementia in these activities which is important for increased emotional well-being and happiness in people with dementia (10).

Prompting technologies and Augmentative and Alternative Communication (AAC) strategies have been used to cue and prompt people with dementia to complete daily activities. However, some of these interventions that involve caregivers, increase caregiver burden by adding constraints on their time and finances and taking away independence and autonomy from people with dementia. Various prompting technologies have been explored such as COACH (Cognitive Orthosis for Assisting aCtivities in the Home) which uses artificial intelligence and computer vision to help guide older adults through the task of handwashing (15). Another example is CIRCA, a touchscreen-based technology that supports caregiver and patient relationships (16). Recent developments in emerging technologies such as mixed reality technology and the Internet of Things (IoT) have provided opportunities to explore scalable and deployable solutions that can greatly improve the everyday lived experiences of people with dementia. For instance, intelligent systems that deploy text and visual prompts using sensors, computer-assisted visions, and artificial intelligence (17) can be used to help users with severe dementia retain their autonomy, improve sleeping patterns, and control incontinence (18).

To maximise the use of mixed reality technologies for positive experiences and impact in the lives of people with dementia, we need to ensure that the interactions with these technologies are designed appropriately, keeping the needs of people with dementia in mind. The perception and action loops should be user-friendly and intuitive for people with dementia to adopt the technology in their everyday activities (19). For this, we need to understand how people with dementia interact with mixed reality technologies and what interaction modalities—types of prompts are easily perceived by people with dementia for subsequent actions (gestures) on the technology. Few researchers have investigated the interactions of older adults with AR technologies through iterative participatory design methods. Jin et al. (20) studied the challenges and experiences of older adults with smartphone AR apps and co-created AR tools to support older adults in the use of AR apps for activities such as setting reminders on the phone. Maxwell et al. (21) and Ullal et al. (22) investigated the use of AR technologies for older adults in long term care settings for social connectedness and collaborative engagement. However, findings for older adults and long term care settings are not transferrable to community dwelling people living with dementia. The activities that people with dementia living in the community engage in and their needs are different to older adults in long term care settings. Thus, this paper describes an observation study with four off-the-shelf mixed reality technologies—two Augmented Reality (AR) and two Augmented Virtuality (AV) technologies to identify prompts that can lead to effective and successful completion of an everyday activity such as learning a new sport or engaging in a new physical activity or going through a rehabilitation regime.

This study is seminal as it paves the way to explore the use of mixed reality technologies as support technologies for prompting people with dementia. This in turn initiates discussions on the accessibility of mixed reality technologies for people with dementia for an inclusive approach to technology design and development.

## 2 Background

### 2.1 Active living and dementia

Early-stage dementia is marked by mild cognitive impairments and moderate cognitive decline. Symptoms could include decreased work performance, increased memory loss, trouble concentrating, problem-solving and managing complex tasks, verbal repetition and trouble carrying out daily activities. These symptoms could deteriorate, affecting everyday communication and activities such as misplacing items, unable to remember recent conversations or events, struggling to find the right words in a conversation, losing track of the day, loss of interest, unwilling to try new things, increased feeling of anxiety, irritability, or depression, trouble remembering names of people and increased trouble planning or organizing. These symptoms and the inability to sequence tasks in an activity make it difficult for people with dementia to engage in activities that promote active living such as cooking, engaging in social conversations, sports, pursuing their hobbies, cultivating new hobbies and skills, etc. This affects their Quality of Life (QoL) which is measured through five key dimensions—well-being in the physical, material, social, and emotional realms and the notion of development and activity (23).

Dementia radically disrupts what can be a normative experience and challenges one's sense of self-worth (24). This impacts everyday life on a holistic level as activities associated with day-to-day life become associated with the allocation of resources like time, money, and energy which are greatly emaciated because of the onset of dementia (25). QoL is often assessed in clinical and diagnostic settings as a measure of ability to carry out Instrumental Activity of Daily Living (IADL). However, people engage in many activities other than IADL such as hobbies, social engagements, rehabilitation routines, rituals and habits that define their QoL.

World Health Organisation (WHO) defines active living as “a way of life in which physical, social, mental, emotional and spiritual activities are valued and integrated into daily living” (3). The Living Well initiative in UK focussed on identifying ways healthcare practitioners, social workers, caregivers and policy developers can support living well in people with dementia. Quinn et al. (26) conducted a study with 1,339 people with dementia to explore experiences of people with dementia with the chronic disease to understand what does living well mean to people with dementia. Majority of the participants wanted to live an engaged and active lifestyle and develop positive relationships with others. Furthermore, studies from many dementia-friendly initiatives and communities have highlighted that active living activities are a key to reduce stigma and exclusion in people with dementia (27).

### 2.2 Activities of active living

Active living can be promoted through activities that keep people with dementia connected with the larger community and help them manage their health and well-being, both physical and mental. Perrin et al. (28) suggest that these activities should be structured and planned to match the needs, abilities, and the dementia journey of people with dementia. The authors proposed the following groups of activities that contribute to wellness experiences: exercise, music & dance, everyday activities, walking, play and games, connecting with nature and water-based exercises. They further detailed how these activities should be adapted to the progression of dementia from the early stage to the advanced stage. For example, everyday activities should include tasks such as shopping, cooking, and housework for people with early dementia. People with dementia should engage in light housework, tidying, sorting, washing up and preparing cold food for mild to moderate dementia. For late-stage dementia, people should participate in activities such as enjoyment of food & drink, taste & smell.

Research has shown that participation in activities that promote active living have significant long-term benefits on cognition, dementia progression and social connectedness. Clark et al. (29) studied effects of Therapeutic Group Singing (TGS) in people with dementia and their caregivers. They found that TGS facilitated communication and connection between people with dementia and their caregivers while helping develop empathetic friendships with other people with dementia. Telenius et al. (5) studied experiences of people with dementia with physical activity, in which they conducted semi-structured interviews with 35 people with dementia. Thematic analysis of the interviews revealed that physical activity gave people with dementia a positive feeling of having mastered a skill and achieved a goal successfully. It gave meaning to their daily routine. For example, every day in the life of people with dementia could start with a morning walk which could be characterised by positive experiences such as having a coffee and a cookie in that special café or meeting friends and acquaintances on the way. Physical activity challenged some people with dementia while for others it was something they looked forward to. Lack of motivation, physical limitations and having to depend on others were the barriers to active living described by people with dementia in the study.

People with mild to moderate dementia experience trouble in sequencing multiple steps in a task. This is the primary barrier to engaging in active living. The impairment affects the ability of people with dementia to execute functions that require planning, sequencing, and attentional control (30). The inability to carry out basic activities in their homes could trigger the need for support from caregivers or could result in the transition to residential care settings, affecting the QoL of people with dementia. Thus, early intervention strategies to prompt and cue people with dementia in the execution and completion of tasks are imperative in response to a diagnosis of early-stage dementia because the impairment affects patients' autonomy and security as they “adjust” to the impairments (31).

### 2.3 Prompting interventions

Neuropsychological rehabilitation approaches to prompt people with dementia involve adaptation of environments and behaviours of individuals around the patient to the cognitive abilities of the patient (32). For example, caregivers often tape off numbers on the number trackpad of a microwave using duct tape, so that people with dementia know which numbers to press to warm up their food. A cognitive prosthesis approach focuses on augmenting the capabilities of the patient to overcome the limitations, rather than doing the tasks for the individual either by a caregiver or a technology replicating the human being (33).

A better understanding of how prompts can be designed to meet the task demands for the level of cognitive impairments in the context of the environment (setting) and the task at hand. Design of assistive technologies to prompt people with dementia to complete everyday activities requires a better understanding of how people with dementia adapt their habits and rituals associated with the activity to cope with the impairment. The activity of tea making for example could be seen as an amalgam of IADL and leisure activity based on what the act of making tea means for the person.

AAC strategies are an area of practice that focuses on addressing individuals who have complex communication disabilities (34). For people with dementia, AAC strategies can allow for greater communication fluency in the face of cognitive impairments and issues with processing memory. Temporal fluctuations in the cognitive experience of people with dementia often lead to problems with task sequencing. Task knowledge is incongruous and prone to error which leads to impairment in completing activities (2). Prompting can help people with dementia stay on track in these cases and help people with dementia follow the subtle cues that help string the tasks together. However, there are contextual factors at play such as level of cognitive impairment, previous familiarity with the task, and environmental comfort (35). Rituals and habits that people with dementia follow while carrying out activities are often given up after the diagnosis, as they adapt to the prompts from the caregivers or the technology. Cognitive prosthesis design of technologies requires designing interactions between humans and technology as a system in synergy with all the people with dementia and all the people socially connected to the patient (33).

### 2.4 Mixed reality technologies for prompting

Mixed reality technologies can offer scalability in terms of adaptations to changes in impairments and individual needs. They offer immersive experiences in the context of engaging with everyday activities. Advances in sensor and wearable technologies including gaze tracking and Neurotechnology will make mixed reality technologies more available in everyday homes, thus making them more deployable (12). With that, the programmability of mixed reality technology will allow them to be adapted to varying applications, contexts and behaviours, thus making them scalable. Mixed reality technologies blend experiences in virtual and physical worlds through augmentation of physical in virtual and vice versa. They fall in the middle of the physical-virtual taxonomy (36). Depending on the type of augmentation, there are two main types of mixed reality technologies in this middle section of the taxonomy: Augmented Reality (AR) augments the physical world with virtual elements and Augmented Virtuality (AV) augments the virtual world with physical objects (37).

The behaviours of people with dementia can be monitored and tracked and the MR system can adapt their behaviour accordingly. For example, AR technology can track gaze, monitor pupil dilations and generate prompts to help people with dementia in everyday activities (38). Leveraging, gaze tracking and eye tracking, prompts can be generated when the user loses track of an activity and their gaze travels elsewhere. Generating self-cued prompts using gaze is known as gaze cueing (39). Previous studies have shown that people with dementia can use gestural actions accurately when prompted by cues (13).

While other prompting studies in mixed reality technologies have provided support for the use of this technology to support IADLs, there are still gaps to address within the research. Accessibility of these technologies and the prompts require investigation in the context of assistive tools for people with dementia in everyday activities. Boyd et al. (8) conducted a usability test with four types of prompts—text, audio, picture, and video to understand the effectiveness of the perception of these prompts for correct actions. people with dementia were asked to take a greeting card, sign it with their name and put the card in an envelope. But mixed reality technologies offer a different experience with the prompts to people with dementia. For example, it has been suggested that designing for immersive experiences in mixed reality technologies requires seamless transitions between physical and digital worlds (40). This could entail experiences such as users being unable to distinguish between real and virtual elements in the design. However, Desai et al. (13) found that such features and experiences make interactions with mixed reality technologies difficult and confusing for people with dementia. They were unable to determine which prompts they could interact with and how. Thus, generating prompts in mixed reality technologies for completing tasks for active living to align with cognitive prosthesis design requires a better understanding of interactions of people with dementia with different modalities of prompts—such as graphics, auditory, text, etc. The objective of this study is to identify the modalities of prompts that provide the required information to people with dementia to carry out correct actions on the technology. The goal was to develop a Framework for the generation of cues for people with dementia in mixed-reality environments.

## 3 Methodology

The study employed the Research through Design (RtD) approach involving User Research and iterative prototyping efforts (41). The user research and insights focused on understanding interactions of people with dementia with off-the-shelf mixed reality technologies to understand how people with dementia perceive cues from technologies and how they respond to those cues with actions performed on the technologies.

### 3.1 User research

An observational study was conducted with twenty-two people with dementia with Alzheimer's disease (AD) and mild cognitive impairment (MCI) (MoCA score = 11 to 25, Age = 63 to 88 years). These participants were recruited for the study through Memory and Company, Alzheimer Society of Durham, and Ontario Shores Centre for Mental Health Sciences in Toronto. The study was approved by the research ethics boards at Ontario Shores Centre for Mental Health and Sciences, University Health Network, and York University.

**Figure 1:**
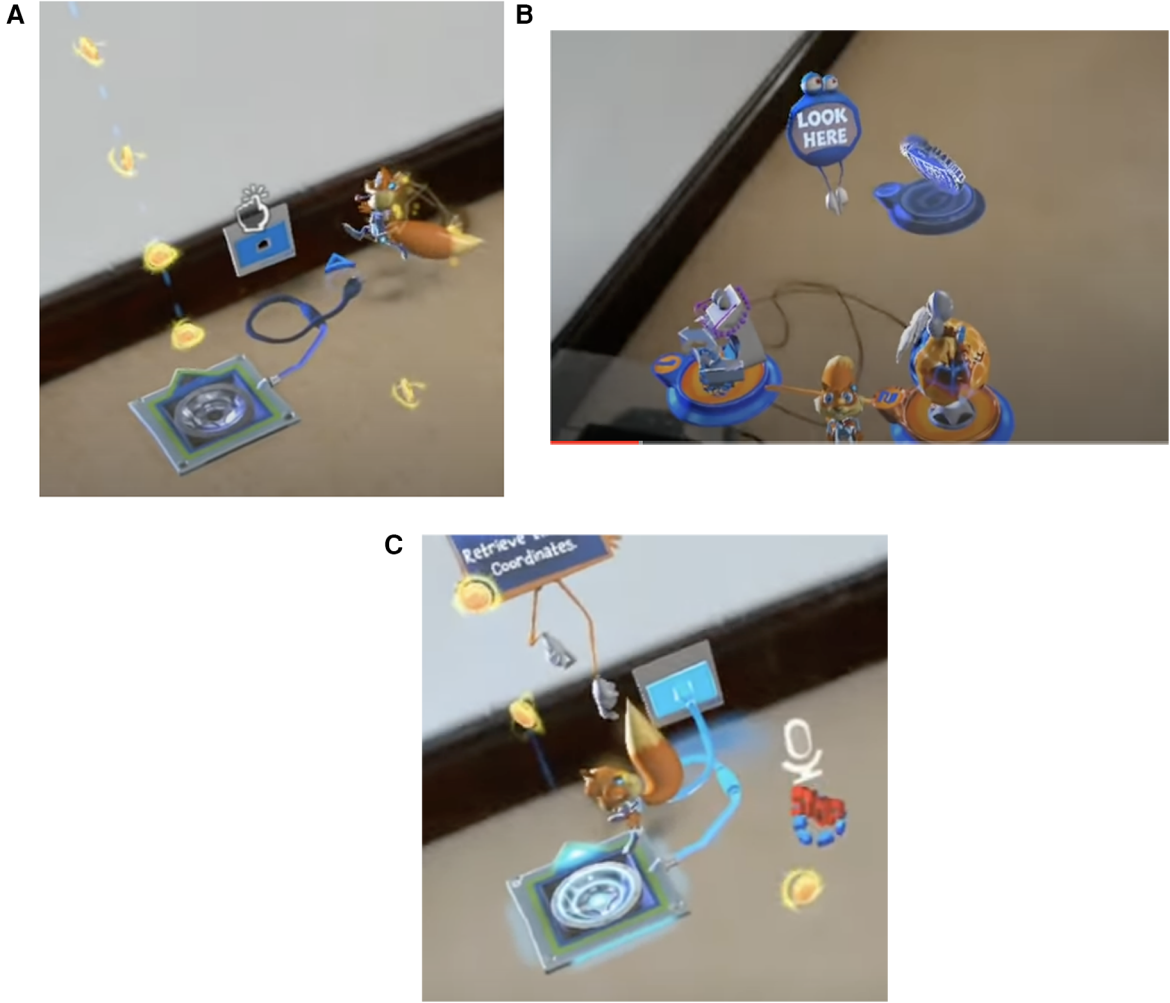
Prompts in the young conker app on HoloLens from microsoft. (**A**) An air tap prompt in the app. (**B**) A text and symbol prompts to cue the user to look at the squirrel named conker. (**C**) Text prompt with fingers pointing to the mission area, a symbol of a microphone to cue the user to speak, a symbol prompt of an arrow to indicate the line of flight for the squirrel.

**Figure 2:**
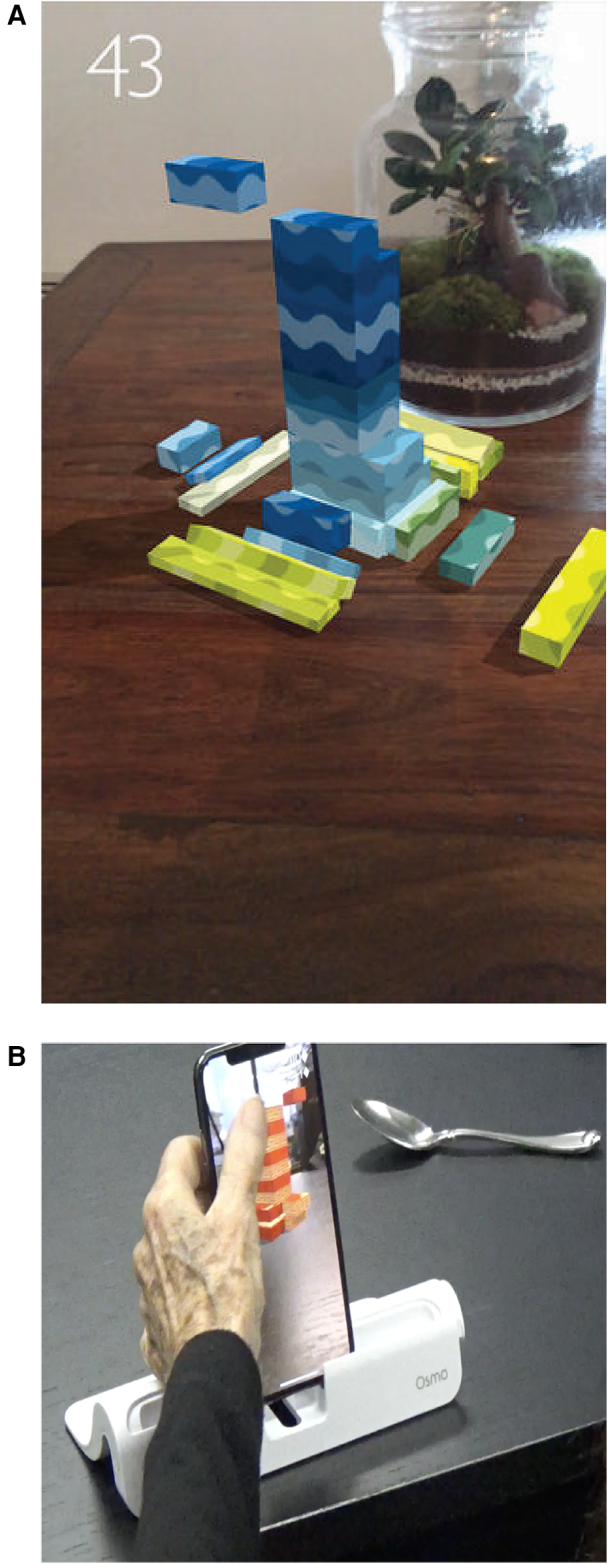
Stackar on iPhone X from apple. (**A**) StackAR app on iPhoneX. (**B**) Visual and feedback prompts in the StackAR app.

**Figure 3:**
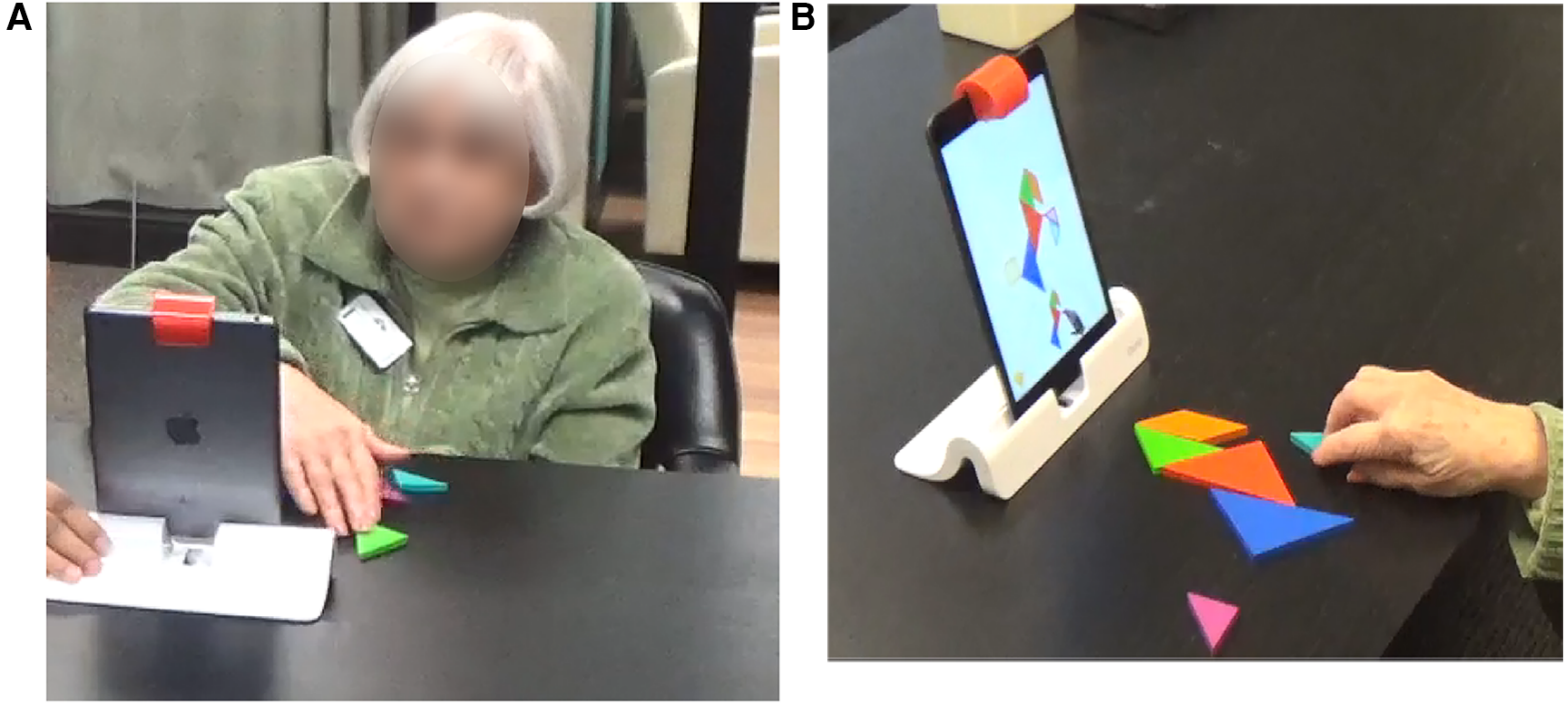
Tangram on osmo from tangible play. (**A**) Front view of a participant playing tangram on osmo. (**B**) Over the shoulder view of a participant playing tangram on osmo, following prompts can be seen here—shape, feedback to an action, flickering visuals (cannot be shown in the image but the blue shape flickers cueing the participant to place the blue block at that spot in the puzzle).

**Figure 4:**
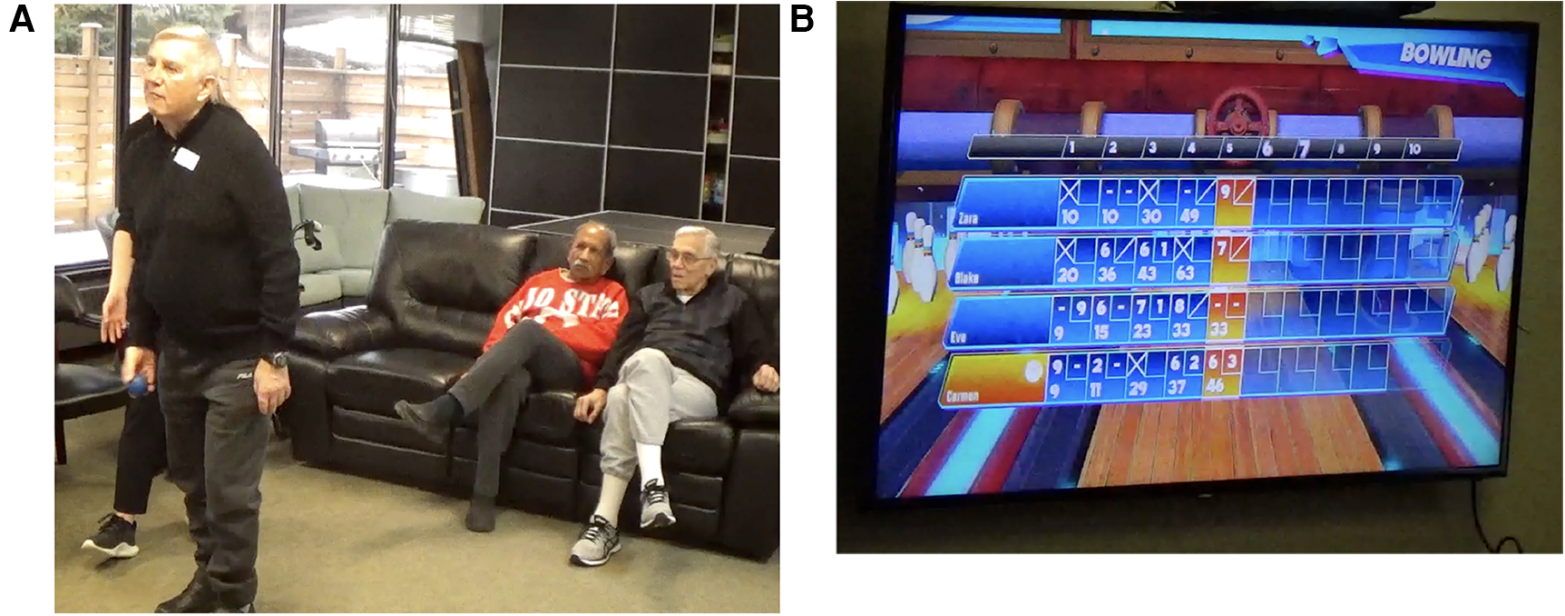
Bowling on Xbox kinect from microsoft. (**A**) Participant using a ball as a prop to play bowling on XBOX kinect while others are watching the game. (**B**) The symbol prompt (arrows) directs the user to throw the ball in a particular direction determined by the arrangement of the bowling pins.

The study was conducted in four sessions on four different days. The cognitive impairment of the participant was recorded using the MoCA assessment tool in the first session, followed by gameplay with four off-the-shelf technologies. Two AR technologies—HoloLens from Microsoft (Figures 1A,B), and ARkit on iPhone X from Apple (Figures 2A,B), and two AV technologies—Osmo from Tangible Play (Figures 3A,B), and Xbox Kinect from Microsoft (Figures 4A,B) were used in the study. Using off-the-shelf technologies provided an effective way to understand how people interact with new and emerging technologies. Playing games with off-the-shelf technologies informs new creative ways to interact with technologies, generating new knowledge about human needs and their perception-action behaviour (42, 43). The emergent aspect in gameplay limits participants' the possibility that participants could plan and anticipate the next steps rationally in the study, thus offering an intuitive platform to understand people's natural behaviour and interactions.

### 3.2 Montreal cognitive assessment (MoCA) session

Participants who showed interest in the study were assessed for cognitive dysfunction using the Montreal Cognitive Assessment (MoCA) (44). MoCA is a cognitive screening tool that assesses short-term memory, visuospatial abilities, executive functions, attention, concentration, working memory, language, and orientation to time and place. It can uncover cognitive impairments associated with disorders including Alzheimer's disease (AD), Parkinson's Disease, Huntington's Disease, Lewy-Body Dementia, Vascular Cognitive Impairment/Stroke, and Frontotemporal Disorders (FTD). In the study, we used MoCA to primarily determine the level of cognitive impairments of the participants, to ensure the eligibility of the participants for the study, that they are in the early stages of dementia diagnosis. It was also used as an icebreaker activity to allow participants and the researcher to get familiar and comfortable in the setting, in preparation for the study sessions.

A full MoCA paper version was used in the study, which is scored on 30 maximum points and administered in approximately 10 min. It consists of 11 activities, which assess visuomotor and visuo-perceptual skills, visuo-constructional skills, visuospatial abilities, word-finding difficulty, semantic memory impairment, short-term memory recall, and the functioning of the frontal lobe to mentally shift between numbers and letters. Clear instructions on the administration of MoCA and scoring guidelines for responses to each activity are provided in the tool. The researcher who administered the MoCA is trained and certified in administering and scoring MoCA.

MoCA scores are interpreted for the severity of cognitive impairment. A final total score of 26 and above is considered normal. A MoCA score of 18 separates MCI from AD, but there is an overlap between the two. The average MoCA score for MCI is 22 (range 19–25), and the average MoCA score for Mild AD is 16 (11–21). MoCA scores between 10 and 17 stand for moderate cognitive impairment, and scores less than 10 are representative of severe cognitive impairment. Participants who scored a MoCA of 11 and above were invited to participate in the study, as we wanted to include both people with Alzheimer's Disease (AD) and Mild Cognitive Impairment (MCI) in the study.

### 3.3 Using play in the study

The remaining three sessions of the study after the initial MoCA assessment session, involved participants playing games using four mixed reality technologies. Out of twenty-two people with dementia who participated in the study, ten participants played Tangram on Osmo. Of these 10 participants, nine played Young Conker on HoloLens and six played StackAR on iPhone X. Fifteen participants played Bowling on Kinect Xbox. The difference in the number of participants playing the three games was because some participants were absent for the day programs at Memory and Company and two participants passed away during the study. The study aims to collect as many interactions of people with dementia as possible with mixed reality technologies and not to compare the technologies. The interactions with different modalities (visual, audio, etc.) were then analysed for successful perception.

We used play as a mode to engage people with dementia in the study, as play creates an environment of responsiveness for the players/participants to yield to the moment (45). Playful settings allow participants to explore all possibilities with the technologies, improvising on the way and creatively identifying ways to interact with the technologies (46). Thus, using play in the research setting allows natural interactions with the technologies. Desai, Blackler, and Popovic (43, 47) in their studies with children co-discovering experiences with physical, digital and mixed reality products found that children not only interacted with technologies naturally but also engaged in discussions with each other. The verbal protocols of think aloud and talk aloud were difficult to incorporate, so these natural conversations provided the required information to support the synthesis efforts.

Playful engagement with technologies is known to stimulate certain cognitive processes in people living with dementia that could allow them to engage in everyday activities such as making a cup of tea and communicating with other people (Augmentative and Alternative Communication). Exergames offer a platform for people with dementia to engage in physical activities in a playful and fun setting. van Santen et al. (48) conducted a controlled study with 73 dyads of people with dementia and their informal caregivers to understand the benefits of exergames. They found that the exergames had a positive effect on people's physical, cognitive, emotional and social functioning and their quality of life. Joddrell et al. (49) studied people living with dementia playing games on tablets and smartphones and developed recommendations and guidelines for accessible touchscreen apps for people living with dementia. They used these guidelines to evaluate various off the shelf apps for phones and tablets and provided the outcomes as a resource for people living with dementia and their caregivers (50). For these reasons, play is an integral part of a dementia management program post-diagnosis. For example, the day program at Memory and Company has games such as puzzles, board games, trivia quizzes, playing cards, crossword puzzles and Bingo built into their daily programs.

The play thus offers a familiar domain in a research study setting for people with dementia to use emerging technologies with confidence and comfort.

The role of play is to create a natural familiar environment for the participants so that the outcomes are not a result of discomfort or anxiety that people with dementia may experience while interacting with emerging unfamiliar technologies. The observation study focussed on the interactions of people living with dementia with cues of different modalities presented by the apps in mixed reality technologies. We made sure that these interactions were not affected by the play by choosing appropriate games for the study. For instance, the games selected were not constrained by time in which the participants were expected to race against time to finish the tasks in the game. During the thematic coding of the data in the data analysis process, care was taken to assess whether the interactions were a factor of gameplay or if they were applicable in other scenarios too.

For participants attending the day program at Memory and Company, the studies could be easily integrated into the daily routine of the day program. For other participants, the study was carried out in the comfort of their homes. Participants played the following games on the four technologies: Young Conker on HoloLens, StackAR on iPhone X, Tangram on Osmo and Bowling on Xbox Kinect. Participants were asked to play the game on the technology for a maximum of 30 min, with an option to stop the game at any time. Before the start of the play with each technology, the researcher provided instructions to the participants on how to use the technology and how to play the games on the mixed reality technology. They were asked to follow the instructions in the app of the game, which are presented as visual and audio cues. Knowing if participants understood the instructional cues, is part of the study.

The researcher prompted the participants when they were seen struggling to figure out how to interact with the technology. These prompts were either verbal cues or physical assistance in the use of the technology or hand gestures. Each play session lasted for a maximum of 60 min. The sessions were video recorded using two cameras: one from behind the shoulder to capture participants' interactions with the technology and responses to the prompts from the technology, and the second from the front to capture the facial expressions and behaviour of the participants.

## 4 Analysis and results

Thematic analysis of the video recordings was conducted using video analysis software from Noldus, Observer XT Version 16.0 (51). We identified two main behaviours in the coding scheme, that this study is primarily focused on—prompts presented by technology and actions/gestures performed by the participants. Each of these behaviours was then described in terms of the modalities of the prompts presented by the technologies and the researcher and the modalities of the interactions by the participants on the technology (Table 1).

**Table 1:** Codebook for perception of multi-modal prompts from the technology and responses to them in a mixed reality environment.

Codes (behaviours)		Description	Examples
Prompts Presented	Visual	These are the cues presented by the technology to the participants for actions to be performed.	Different shapes with and without colour information, text, animations of graphics elements, cartoon characters, statistics (numerical and graphs), researcher intervening through physically helping the participants or through verbal instructions or through gestures, human voice over, music tones, cartoon voice, etc.
	Interventions from the researcher		
	Sound		
Actions/gestures from people with dementia		These are the actions performed by the participants in response to the cues presented. Only correct and successful actions were coded.	Verbal responses/speech, touchscreen interactions, using gestures such as air tap, gaze, head movements, tangible interactions, etc.

We exported the coded data to Excel to measure the behaviour of people with dementia with the technologies. In this study, we were specifically interested in measuring the interactions with mixed reality technologies. The metrics for behaviour measurement depend on how the behaviours are recorded, but the prominent metrics are frequency and duration of behaviours (52). We focused on the frequency of behaviours in this study, as we were interested in how often people with dementia perceived the prompts successfully. A successful perception for this study is considered when people with dementia respond to a prompt from the technology or the researcher with a correct action/gesture.

There was a total of seven hundred and ninety-four (794) behaviour events coded. Fourteen (14) types of prompts (shown in Table 2) were identified which were either visual, sound or intervention modality while five (5) types of actions/gestures (shown in Table 3) were coded. Since we were only interested in measuring the successful perception of the prompts, we only measured behaviours where the code “Prompts Presented” was followed by the code “Actions/Gestures from people with dementia”. Filtering out behaviours not relevant to the investigation resulted in two hundred and forty-three (243) behaviour events of “Prompts Presented” that were successfully perceived. Two hundred and seventy-nine (279) “Actions/Gestures by people with dementia” followed the “Prompts Presented”, suggesting that some prompts were followed by more than one action/gesture. We calculated the total frequency of occurrence of each of the twenty-one types of prompts that were identified for the behaviour code “Prompts Presented”. We also calculated the frequency of the occurrence of the behaviour code “Actions/Gestures of people with dementia” following these prompts (see Table 2). This allowed us to estimate the probability that the prompts were followed by a correct action/gesture, thus indicating the probability of being successfully perceived by people with dementia in the Mixed Reality environment.

**Table 2:** Probability of successful perception of prompts by people with dementia in mixed reality.

No.		Types of prompts for the behaviour—“Prompts Presented”	Total Frequency of prompts presented by the technology	Frequency of the behaviour “Actions/Gestures of people with dementia” following the prompt	Probability of successful perception of the prompt
1	Visual Prompts	Statistics	80	0	0
2		Symbols	133	93	0.69
3		Shapes	367	140	0.38
4		Text	273	157	0.58
5		Animated Symbols	142	68	0.48
6		Animated Characters	139	34	0.24
7		Flickering Visuals	64	11	0.17
8	Sound Prompts	Human Voice	135	100	0.74
9		Tonalities and Timbres	156	19	0.12
10		Voice of a Cartoon Character	86	48	0.56
11	Feedback	Feedback to an Action	205	126	0.61
12	Intervention	Gestures	77	2	0.03
13		Speech	518	109	0.21
14		Physical	168	11	0.06

**Table 3:** Types of actions/gestures with the mixed reality technology by people with dementia identified in the study.

No.	Types of actions/gestures of people with dementia	Description
1	Speech	people with dementia interacting with the technology by talking with it.
2	Embodied Actions	This included interactions such as object manipulation, body movements that are part of the actual game (tennis, bowling, etc.)
3	Gaze	Using eye gaze by moving head in a particular direction to control the virtual elements in the game.
4	Gestural	This included hand gestures such as air tap, bloom, etc.
5	Touchscreen	Interactions with screens such as swipe and tap

**Figure 5:**
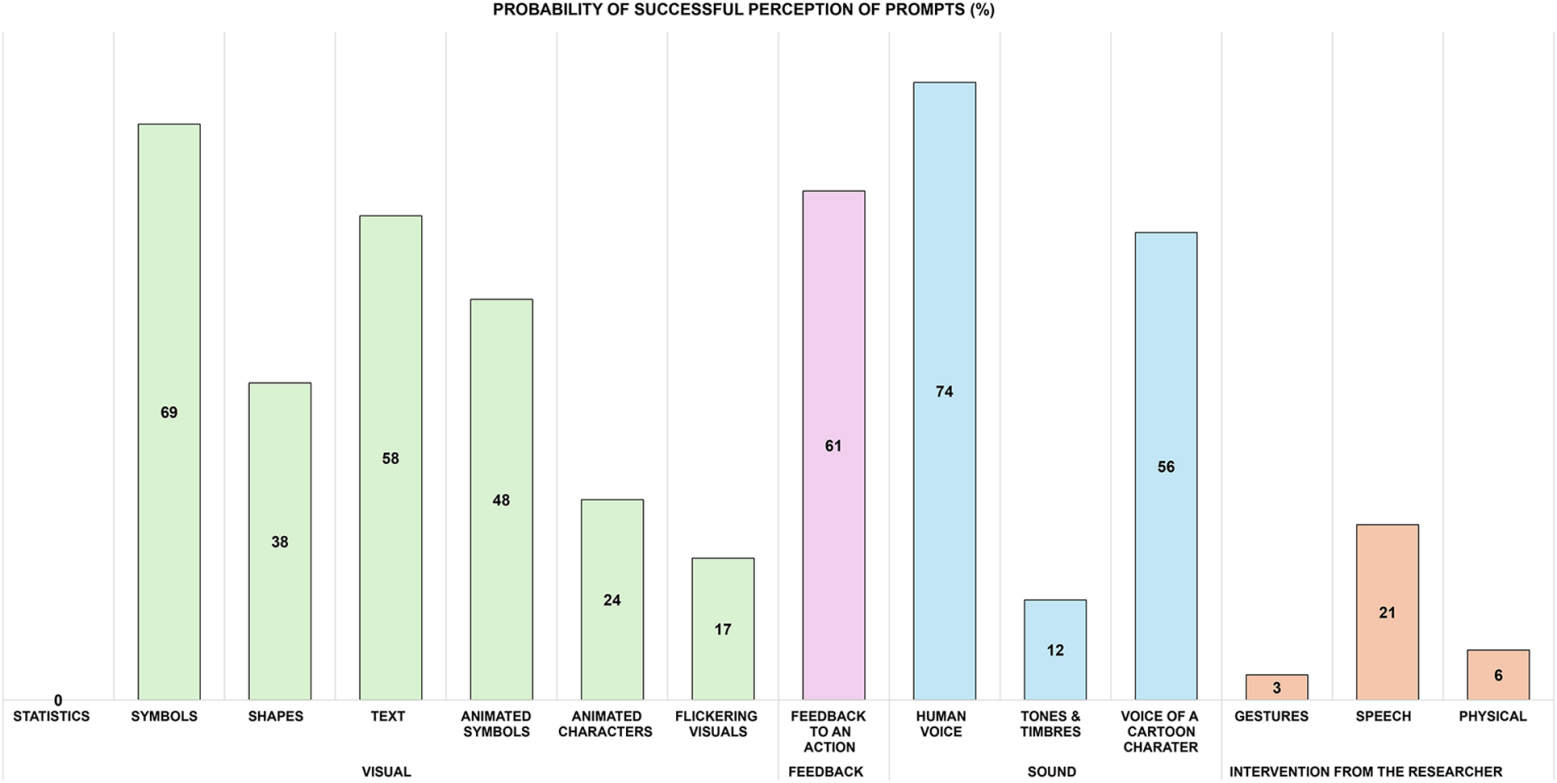
Clustered Bar plot of probability of prompts successfully perceived by people with dementia.

**Figure 6:**
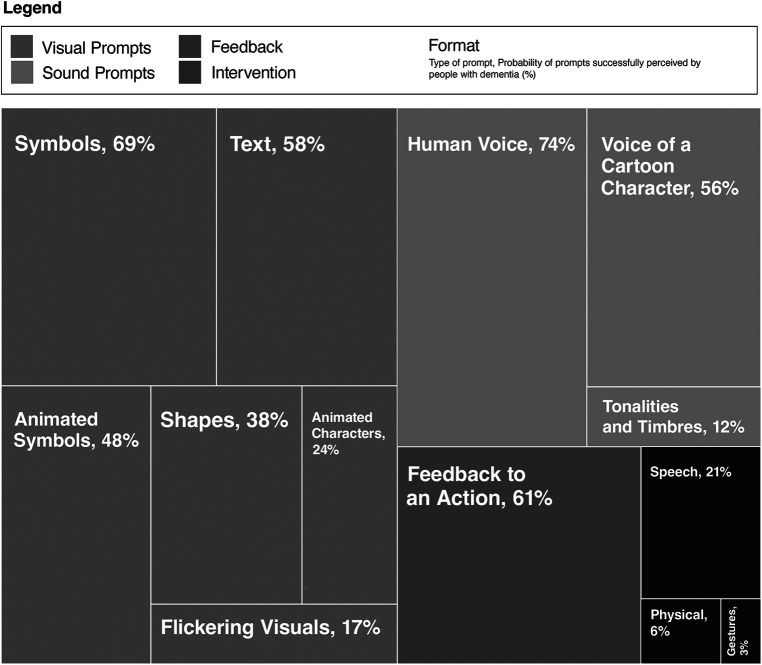
Four-level treemap showing the hierarchy of the probability of prompts successfully perceived by people with dementia.

The probabilities are shown in Table 2 and the bar plot (Figure 5) for a comparison of the perception of the prompts in people with dementia. A treemap was used to plot the hierarchical visualisation of the probability of the prompts successfully perceived by people with dementia (Figure 6). The plot is split into rectangles, that are sized and ordered by the probability of the prompts successfully perceived. Since there are three categories of prompts identified in the study, a three-level treemap was created. The three categories of the prompts—Visual Sound and Intervention from the Researcher, are visualised as rectangles containing other rectangles representing the probability of the prompts in the category. The two plots (bar and treemap) allowed us to compare the perception of the prompts, informing a framework for design of prompts in an interactive interface for people with dementia in a mixed reality environment.

The results suggest that people with dementia were unable to understand the statistical information presented by the technology. This included bar graphs, pie charts and numerical raw data. Symbols, graphics and text prompts were most successfully perceived by people with dementia; however, they had some trouble responding to the animated symbols and graphics. Shapes were moderately perceived. The most difficult to perceive after statistical information were flickering visuals and the use of animated characters, for example, movements of design elements such as text, graphics symbols and characters.

The voice of a human was the most perceived, not only within types of sound prompts but also in comparison to visual prompts. The voice of a cartoon character (such as Mickey Mouse created by Walt Disney and Ub Iwerks) was moderately perceived, and the tones of varying frequencies and intensity (tonality and timbre)) were difficult to understand for people with dementia. When it came to interventions from the researcher, the verbal prompts were most perceived. Feedback to action received correct responses from people with dementia and has a high probability that they could be perceived.

## 5 Discussion

The results and findings suggest that some cues are easily perceived by people with dementia than others, the human voice being the most perceived while the information in the statistical graphs was not understood by people with dementia. The most successful prompts were the cues that were easy to understand and those that exerted less cognitive overload to decide actions to be performed on the interfaces. This is also the premise for designing user-friendly interfaces that are intuitive and easy to use (53). Providing feedback to every action carried out by people with dementia helps reduce this cognitive overload, which explains the successful perception of these prompts. It is also a natural way in which how humans interact and communicate. They anticipate the presence of cues to help in deciding their next course of action. The coupling between perception and action governs how information is processed by people and how they adapt their behaviours accordingly (54). This means that the perception-action coupling needs to be “tight” for successful information processing and interactions with interfaces. The feedback prompt to action helps keep the perception-action loop going to allow adaptive and emergent behaviours from the users (36).

According to Fischer (55), “the challenge for future human centred computer systems is not to deliver more information to anyone, at any time, and from anywhere, but to provide the right information, at the right time, in the right place, in the right way, to the right person”. Thus, the interactions with computing systems such as mixed reality technologies are determined by the context—the people involved, their familiarity (42), abilities (56), the objective of the interaction and the time and place of interaction (57). Thus, the prompts including the feedback prompts should be designed for the needs and abilities of people with dementia. The study observed people with dementia in the context of play and we identified prompts that are easy to perceive and act on for people with dementia, focusing on the context of user abilities and cognition.

### 5.1 Perception of auditory prompts

The human voice as a prompt was most successful in generating correct actions. It is a natural form of interaction and is familiar to people with dementia as the cues from caregivers and family members are mostly instruction-based. Clinical trials have shown that promoting conversations in people with dementia is an effective way to tackle social isolation and further cognitive decline (58). Previous work on conversational agents and robotics has explored the use of voice as a natural mode of interaction in people with dementia (59–61). Efforts have been put into making robots as human-*like* as possible to facilitate social interactions by enabling conversations in people with dementia in addition to effective communication modalities such as gestures and facial expressions (62). Virtual AI assistants such as Amazon Echo and Google Home have been trialled in the homes of community-dwelling people with dementia. Beh et al. (63) evaluated Amazon Echo with people with dementia to manage their everyday tasks, with Alexa providing voice prompts. They found that the virtual assistants were effective when people with dementia saw utility in the use of the technology, which Desai et al. (14) also found in their study with older adults. People with dementia were able to use the virtual assistants successfully to build routines, ask for time, seek help with crosswords and create reminders. However, future studies are required to assess the quality and nature of conversations with technologies that work successfully in prompting people with dementia. For these technologies to mimic a buddy or a caregiver, the conversations need to be natural. The technologies could learn and adapt from their conversations with the person with dementia which humans (caregivers) often find difficult to do because of the overburdening with caregiving and other responsibilities.

The mixed reality technologies also presented other forms of sound prompts such as the voice of a cartoon character and tonalities and timbres (music with varying pitch, tone and quality). While the human voice was most successful in perception and subsequent action, the other forms of sound prompts were less to moderately successful. People with dementia also expressed dislike towards the voice of the cartoon character. Participant P1 while playing Tangram on Osmo said, “This is childish. How do I make it stop talking”. The music prompts presented by all mixed reality technologies—Osmo, HoloLens, ARkit on IphoneX and XBOX Kinect, were often not noticed by people with dementia.

### 5.2 Perception of visual prompts

Visual symbols and graphics can be successful with people with dementia if they are designed in a way that people with dementia do not feel the cognitive overload in processing their meaning. Picture symbols have been successfully used in the Talking Mats project to facilitate communication between caregivers and people with dementia (64). Symbols such as an exclamation representing danger/attention suggesting that the user should stop doing what they are doing or they should not go beyond a certain point, a tap icon indicating that the user should select an option by tapping their finger on it, arrows indicating direction and a microphone means users should speak to interact with the interface, were quickly understood and successfully interpreted. However, metaphorical symbols such as coins collected in a pot with a jingling sound representing successfully finishing a level in a game or winning the game were not understood. Participant P20 said, “What has coins to do with it (winning the game)?”. Both relevance theory (65) and conceptual metaphor theory (66) suggest that metaphors are natural and fundamental to human cognition. However, they do impose additional effort in making the connection between the symbol, the representation, and the context. Metaphors require two steps for the users to interpret their meaning—understand the reference and the intention of the design (67). In the example visual of collecting coins, the user must understand the reference of receiving coins to winning or task completion and then make an inference that the designer/game is rewarding the user. This takes more time and effort, and if the user fails in the first step, they cannot make meaning out of it. For people with dementia, this could mean confusion in the process of interacting with mixed reality technologies, but this also affects their confidence in the activity the technology is facilitating. Additionally, different people could comprehend the metaphor differently. Considering this, the metaphors should tap into the natural affordances of materiality and embodied actions or references to these (36), familiarity and previous knowledge of people with dementia (42) for intuitive interaction with the prompts.

Graphics and symbols with fast movements like animations and flickering graphic elements (motion graphics) are difficult for people with dementia to understand. This is partly because it causes discomfort to the eyes and the cognitive processes. Movement changes the state of the elements before people with dementia can interpret and understand the meaning of the visuals, which ultimately results in them avoiding looking at these elements. People with dementia mostly ignored these types of prompts, claiming that they did not notice them. Animations and movement in visual prompts should be subtle and with slow movements, allowing people with dementia enough time to see and interpret the meaning of the prompts.

Text prompts were very effective both in terms of successful perception of the prompts and engagement. people with dementia read all text prompts from start to end, however long the sentence was. However, care should be taken in writing text-based prompts; using language that is easy to understand and the use of metaphors should be avoided. For example, people with dementia in the study could not understand the meaning of the word “flip” in a text prompt in the game of Tangram with Osmo mixed reality technology. The prompt was a suggestion to turn the block over in the puzzle. When the researcher prompted the participant with the words “Turn Over”, they immediately turned the block over.

Dementia especially Lewy-Body dementia and dementia caused by Parkinson's Disease, affects visuospatial abilities and skills, due to which people with dementia have difficulty understanding visual information and interpreting spatial relationships. Thus, they experience challenges differentiating between different shapes, scales, sizes and their spatial orientation and placements. However, this study reported an unusual finding concerning the perception of shapes and their spatial information. People with dementia could successfully identify different shapes of different sizes and colours in the game of Tangram on Osmo mixed reality technology, some shapes were filled with colour while some had coloured outlines only. People with dementia could also translate 2-dimensional information on the tablet screen to actions in the 3-dimensional space and vice versa. They could differentiate whether the prompts represent horizontal or vertical orientation. It is our understanding that this is because the prompts were generated as feedback to an embodied action of manipulating objects in the physical space (36). People with dementia were using the materiality and affordances of the blocks and the puzzle layout to arrange the pieces together. Participants tried out the spatial layout of blocks and their orientation. The feedback to this object manipulation was displayed on the screen, suggesting whether the block placement was correct and what the next corrective step should be in the task of solving a puzzle. Most of the participants had difficulty using the gesture of “bowling” with the XBOX Kinect mixed reality technology. There could be multiple reasons for this—lack of prior experience playing bowling, due to the cognitive impairment of dementia or due to lack of appropriate prompts to help people with dementia complete the tasks. However, when the researcher intervened and handed the participants a plastic ball and asked them to swing their arms, the participants were successful in carrying out the required bowling action in the game. Thus, using props to facilitate embodied actions with appropriate feedback prompts to the actions could facilitate the correct and successful perception of prompts in people with dementia.

### 5.3 Actions

Non-verbal forms of interactions such as gestures were more effective than those that required language and semantic forms of cognitive processing. People with dementia were observed successfully interacting with mixed reality technologies with unfamiliar gestures such as mid-air gestures in three-dimensional space in HoloLens. Astell et al. (68) also reported that familiarity does not play a significant role in using gestures in interactive technologies.

People with dementia were frustrated and confused when HoloLens could not detect their gestures because they were not within its field of view. A similar observation was also noted with Xbox Kinect. When people with dementia were not standing while playing within the field of view, the technology did not detect the bowling gestures of the participants.

Regular reminiscence of new learning helps people with dementia to glue the tasks together. The technology should be designed to prompt the learnt gestures to people with dementia every time they need to use them again. The researcher prompted the participants in the study using gestures and speech to use the “Bloom” gesture while interacting with HoloLens. Jin et al. (20) suggested that trial and error learning helps older adults to effectively use AR apps with subsequent use of AR apps with reduced physical and cognitive strain. However, trial and error strategies should be appropriately supported in technologies designed for people with dementia. The tangram puzzle in Osmo mixed reality technology, discussed above in section 5.2, does not require any reminiscence prompts to re-train people with dementia on the gesture of object manipulation as they learn through trial and error methods. However, this is facilitated by the material affordances offered by the objects themselves (36). Thus, the use of embodied gestures could offer intuitive ways to interact with mixed reality technologies.

Speech based interactions were difficult for people with dementia. Most participants used gestures before they spoke to interact with the technology as well as to communicate with the researcher, irrespective of whether the technology supported the gesture or not.

**Figure 7:**
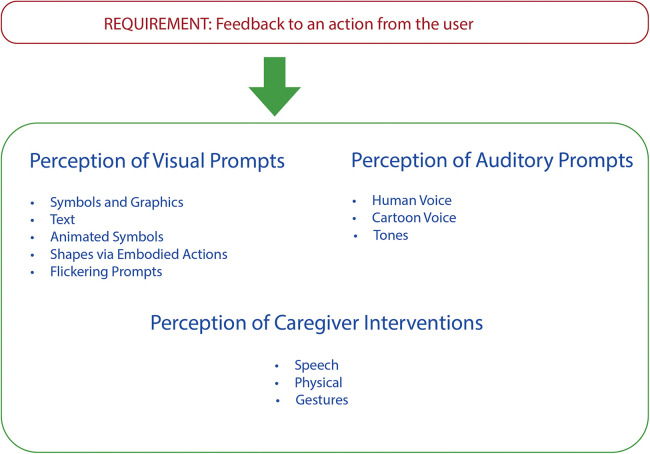
Design guidelines for generation of cues for people with dementia in mixed reality environments.

Based on the above discussion, we present design guidelines and recommendations for the generation of prompts for people with dementia in mixed reality environments, as shown in Figure 7. These guidelines are aimed at supporting designers in developing mixed reality based prompting technologies for people with dementia. We are currently using these guidelines to develop numerous prototypes for a range of everyday activities such as making a cup of tea and checking the weather for planning for the day. These applications are being developed on web portals, smartphones and mixed reality technologies—HoloLens, ARkit and Quest Pro using user task flow analysis, Generative AI and interface development on Unity OpenXR and MRTK3. The evaluation of these prototypes on different platforms and for varying activities will further inform the design guidelines.

## Data Availability

The datasets presented in this article are not readily available because the dataset represents qualitative data of human interactions with technologies. Consent from participants was not obtained to share the data as it is. So we cannot share the raw data of the participants. Requests to access the datasets should be directed to desais@yorku.ca.
